# A Retrospective Analysis of Emergency Obstetric Hysterectomy: A Life-Saving Intervention

**DOI:** 10.7759/cureus.46758

**Published:** 2023-10-09

**Authors:** Aruna Verma, Garima Sharma, Monika Kashyap

**Affiliations:** 1 Department of Obstetrics and Gynecology, Lala Lajpat Rai Memorial (LLRM) Medical College, Meerut, IND

**Keywords:** obstetric hemorrhage, postpartum hemorrhage, exploratory laparotomy, maternal mortality, emergency obstetric hysterectomy

## Abstract

Introduction

Emergency obstetric hysterectomy (EOH) is a lifesaving procedure that plays a critical role in managing life-threatening obstetric emergencies. In our study, we sought to evaluate the incidence, indications, risk factors and maternal as well as fetal outcomes associated with EOH. Also, the study was conducted to review the operative experiences and trend of emergency hysterectomies done for various indications over a period of two years in our tertiary care center.

Methods

The present hospital-based retrospective analytical study was conducted in the Department of Obstetrics and Gynecology, L.L.R.M. Medical College, Meerut, between March 2021 to February 2023. All women who delivered within or outside the institute undergoing obstetric hysterectomy were included in our study. Out of a total of 7743 deliveries, 46 EOH cases were found. The data of these 46 EOH deliveries were collected and analyzed after issuing ethical clearance by the Institutional Ethical Committee of L.L.R.M. Medical College, Meerut.

Results

During the two-year study period, 46 EOH were performed out of 7743 deliveries making an incidence of 0.504 EOH per 100 deliveries. Most of the cases were of multiparous women in the age group of 25 to 35 years (78%). The majority, 43.5% cases belonged to placenta accreta spectrum (PAS), followed by ruptured uterus (30.5%) and postpartum hemorrhage (PPH) (26%). The most frequent preoperative complication seen in EOH was severe anemia (29, 63%). Intraoperative bladder injury was seen in four cases, along with one case of ureteric injury. Postoperatively, cases were shown to have acute hemorrhagic shock (54.3%), disseminated intravascular coagulation (DIC), septicemia, wound infection, acute renal failure (ARF), hepatic encephalopathy and psychosis. Four maternal mortalities were observed in our study.

Conclusion

EOH is a necessary operative procedure in many obstetric emergencies including PAS and PPH. Despite intra-operative risk and possible postoperative complications, it remains a potentially life-saving procedure. Thus various surgeries for PAS and PPH should be the integral part of postgraduate trainings to save the lives of mothers and to reduce the maternal mortality.

## Introduction

Antepartum hemorrhage (APH) and postpartum hemorrhage (PPH) still are the most dreadful and unforeseen complications in obstetrics. In extreme cases, when all other measures fail to control bleeding and preserve the life of the mother, emergency obstetric hysterectomy (EOH) becomes a necessary procedure. EOH is defined as the removal of the uterus either at the time of caesarean section (CS) or following vaginal delivery (VD) within the puerperium, and it is usually performed in the face of life-threatening obstetric hemorrhage [1].

Over 500,000 women die each year due to complications of pregnancy and childbirth, a number that has remained relatively unchanged since 1990, when the first global estimates of the burden of maternal mortality were developed [2]. Hemorrhage due to uterine atony, and placenta accreta spectrum (PAS) are still the causes of maternal death in developing countries [3]. Hysterectomy was originally employed in Obstetrics a hundred years ago as a surgical attempt to manage life-threatening obstetrical hemorrhage and infection [4]. Nowadays, it is generally performed as a lifesaving procedure in cases of ruptured uterus, resistant PPH, PAS and uterine sepsis.

Over the years, the incidence of EOH due to uterine atony has declined from 42% to 29% because of the use of uterotonics and conservative surgical procedures, but the incidence of abnormal placentation has increased from 25% to 41% due to increasing number of CSs. Also, performing an EOH on a vascular gravid uterus needs expertise, and the maternal outcome greatly depends on the timely decision, the surgical skills and the speed of performing surgery. So, despite being the last resort to save a mother’s life, the reproductive capability of women is also sacrificed. Hence, after assessing the incidences, risk factors, indications and outcomes, this paper introduces the concept of EOH as a critical intervention that can save lives in obstetric emergencies, shedding light on its significance and providing an overview of its key aspects, as no such type of study was conducted at our tertiary care centre previously.

## Materials and methods

Study setting

The present hospital-based retrospective analytical study was conducted in the Department of Obstetrics and Gynecology, L.L.R.M. Medical College, Meerut. It is the major government referral center as well as a teaching and training institute in western Uttar Pradesh, India.

Study population

All cases of emergency obstetric hysterectomy (EOH) between March 2021 and February 2023 were included after fulfilling inclusion and exclusion criteria. EOH is defined as a hysterectomy done after 24 weeks gestation and within six weeks of delivery [5, 6]. Inclusion criteria included all women who delivered in the hospital between March 2021 and February 2023 after 24 weeks of gestation, and who underwent hysterectomy for obstetric indications at the time of delivery or subsequently within the defined period of puerperium (42 days).

All women who delivered outside the hospital and were referred for obstetric complications meriting a hysterectomy and fulfilling all the above conditions were also included in the study. Women who delivered before 24 weeks of gestation, undergoing hysterectomy for gynecological reasons (e.g., sterilization or cancer) or outside the stipulated time of 42 days post-delivery only referred for further management were excluded from the study.

Data collection

Data were collected retrospectively from March 2021 to February 2023 from the central record section of L.L.R.M. Medical College, Meerut and subsequently all the data were reviewed and analyzed. Extracted information from medical records included socio-demographic characteristics (age, antenatal visits, booking status, age), obstetric history (parity, previous deliveries), type of delivery (vaginal or caesarean and their indications), clinical indicators (uterine rupture, intractable PPH, PAS), type of EOH, distribution of blood products transfused and other possible outcomes of EOH [anemia, bladder and ureter injury, shock, sepsis, disseminated intravascular coagulation (DIC), wound infection, acute kidney injury (AKI), maternal and fetal mortality]. We found a total of 7743 deliveries in the selected time frame. Out of these 7743 deliveries, 46 had undergone EOH.

Data analysis

The collected data of these 46 EOH deliveries was entered into the predesigned working proforma. All the information extracted was filled onto the Microsoft Excel Spreadsheet, and analyzed by simple descriptive statistics performed, using IBM SPSS Version 25.0 (IBM Corp., Armonk, NY, USA) and described with the help of tables. Mean as well as standard deviation were used for categorical data and percentages were used for continuous variables. The main focus was kept on indications and surgical outcomes of EOH.

Ethical considerations

The ethical clearance for this study was issued by the Institutional Ethical Committee of L.L.R.M. Medical College, Meerut (IEC No./SC-1/2023/1966). The information of all patients was coded to ensure confidentiality.

## Results

During the two-year study period, a total of 7,743 deliveries were performed, of which 46 underwent EOH yielding an incidence of 0.5941 per 100 deliveries. Additionally, the incidence rates specifically for EOH after vaginal delivery and EOH after cesarean are provided as 0.03 per 100 deliveries and 0.5553 per 100 deliveries, respectively (Table 1).

**Table 1:** Incidence of emergency obstetric hysterectomy EOH: Emergency obstetric hysterectomy

Statistical data (within a given duration)	Number	Incidence
Total births	7743	-
Number of vaginal deliveries	3021	-
Number of cesarean deliveries	4722	-
Total Number of EOH	46	0.5941 per 100 deliveries
Number of EOH after vaginal delivery	3	0.03 per 100 deliveries
Number of EOH after cesarean delivery	43	per 100 deliveries

The majority were multiparous women in the age group of 25 to 35 years. They constituted over 78% of cases, out of which 40% of women were of parity 2 and 40% of parity ≥3. No primigravida woman was encountered during our study period (Table 2).

**Table 2:** Distribution of cases by age and parity

Age(years)/parity	P1	P2	P3	P4	P5 or >5	Total
<25	3	2	2	0	0	7
25-30	3	9	5	1	1	19
30-35	4	6	2	1	4	17
>35	0	1	1	1	0	3
total	10	18	10	3	5	46

Most of the cases (60.9%) belonged to the lower-middle class, and 93.5% were unbooked. The mean gestational age of the mothers who underwent EOH was 36.5 weeks, with a standard deviation of 3.422. Thirty-seven percent of cases had previous normal deliveries, while 12 cases were of previous 1 cesarean and 17 cases of previous ≥2 cesareans representing 63% of the total. Regarding mode of delivery, three patients underwent vaginal delivery (6.5%), 33 cesarean (71.7%) and 10 cases required exploratory laparotomy (21.8%). Nine exploratory laparotomies were done due to ruptured uterus and one was done in view of uncontrolled atonic PPH developed after VD. Among the cesarean cases, 12 were performed due to a previous cesarean delivery in which three cases were found to have scar dehiscence followed by rupture, and the rest cases had PAS and PPH. Two cases were related to already diagnosed cases of placenta accreta, and 10 cases were associated with APH. Obstructed labor was seen in two cases, out of which one case was complicated as intractable PPH and another one presented as a ruptured uterus. Seven cases were attributed to other obstetric indications in which one with transverse lie was found to have posterior wall rupture. However, nine cases of preoperatively diagnosed rupture uterus and one case of intractable PPH after vaginal delivery led to exploratory laparotomy (Table 3).

**Table 3:** Distribution of cases according to various demographic and obstetrical factors N: Total number of cases; SES: Socioeconomic status; APH: Antepartum hemorrhage; PPH: Postpartum hemorrhage; SD: Standard Deviation

Maternal factors	N=46	Percentage
SES		
Upper/Upper middle/Upper lower	18	39.1%
Lower middle/Lower	28	60.9%
Booking status		
Unbooked	43	93.5%
Booked	3	6.5%
Gestational age	Mean- 36.57 (SD-3.422)	-
Obstetric factors		
Previous normal deliveries	17	37%
Previous 1 cesarean	12	26%
Previous ≥2 cesarean	17	37%
Mode of delivery		
Vaginal	3	6.5%
Cesarean	33	71.7%
Previous cesarean delivery	12	26
Placenta accreta	2	4.3
APH	10	22
Obstructed labour	2	4.3
Other Obstetric indications	7	15.2
Exploratory laparotomy	10	21.8%
For rupture uterus	9	-
For PPH	1	-

Of the 46 cases of EOH studied, 39 (84.8%) of deliveries were institutional, whereas seven (15.2%) of patients were delivered outside the hospital and were later referred for further management. PAS, uterine rupture and intractable PPH were the chief indications for EOH in our study. The majority 43.5% cases belonged to the PAS in which 10 cases were due to a previous cesarean section, seven cases were associated with APH and placenta previa, two cases were related to APH without placenta previa, and one case was due to retained placenta. About 30.5% of the total EOH cases were done for rupture uterus. Nine cases were shown to have rupture uteri because of their grand multiparity, three cases were associated with dehiscence of a previous scar, and one case was ruptured due to obstructed labour; however, one patient had rupture uterus in a grand multipara with a transverse lie presentation. Among all cases of PPH, seven cases were due to atonic uterus (inadequate contraction of the uterus), three cases were related to traumatic causes (such as rectus sheath hematoma, broad ligament hematoma and bladder wall tear along with hemoperitoneum) and two cases involved both atonic and traumatic causes of PPH. The type of EOH was total and subtotal hysterectomy in 39.1% and 60.9% of cases, respectively. Total hysterectomy was performed mainly for the cases of low-lying placenta, PAS or where removal of the cervix was mandatory to achieve hemostasis (Table 4).

**Table 4:** Indications of Emergency Obstetric Hysterectomy N: Total number of cases; APH: Antepartum hemorrhage; PPH: Postpartum hemorrhage

INDICATIONS	N=46	Percentage
Placenta Accreta Spectrum	20	43.5%
Post cesarean	10	-
APH with placenta previa	7	-
APH without placenta previa	2	-
Retained placenta	1	-
Rupture uterus	14	30.5%
Grand multipara	9	-
Dehiscence of the previous scar	3	-
Obstructed labour	1	-
Grand multipara with transverse lie	1	-
PPH	12	26%
Atonic	7	-
Traumatic	3	-
Both	2	-

The most frequent preoperative complication seen in EOH was severe anaemia (29, 63%). Others were intrauterine death of fetus, DIC, thrombocytopenia, hypertensive disorders, gestational diabetes mellitus (GDM), AKI, and Rh-negative pregnancy seen in >40% of cases. Thirty-one patients (67.4%) presented with hemorrhagic shock at the time of arrival. Intraoperative complications include bladder injury seen in four cases, followed by repair, while in one case ureter was also injured along with bladder injury corrected by bladder repair and DJ stenting. In 95.7% of cases, ventilator support was needed with added maternal morbidity. Postoperatively, 54.3% of cases were shown to have postoperative hypovolemic shock, specifically acute hemorrhagic shock, DIC was seen in nine cases, four cases of septicemia, two of wound infection and 15.2% had others (such as acute renal failure, hepatic encephalopathy, psychosis). The average duration of hospital stay was 9.89 days, with a standard deviation of 3.956. There were an overall four maternal mortalities, representing 8.7%, one fetal mortality and 18 intrauterine fetal deaths. Almost all women received blood products with a median of 3 units (range 1-6) of packed red blood cells (PRBC) and a median of 4 units (range 0-8) of fresh frozen plasma and 8 units of platelets transfused in one patient with thrombocytopenia (Table 5).

**Table 5:** Perioperative complications N: total number of cases; IUD: Intrauterine death; DIC: Disseminated intravascular coagulation; ARF: Acute renal failure

Pre-op complications	N=46	Percentage
Anaemia	29	63%
Diagnosed IUD	5	10.8%
Hypertension disorders	3	6.5%
Others	16	24.7%
Patients presented with shock	31	67.4%
Intra-op complications		
Bladder injury	4	8.6%
Ureteric injury	1	2.1%
Need for ventilator support	44	95.7%
Post-op complications		
Shock	25	54.3%
DIC	9	19.6%
Septicemia	4	8.7%
Wound infection	2	4.3%
Others like ARF, hepatic encephalopathy, psychosis	7	15.2%
Duration of hospital stay (days)	mean=9.89±(SD=3.956)	-
Maternal Mortality	4	8.7%
Fetal outcome		
Live	27	58.7%
IUD	18	39.1%
Mortality	1	2.2%

All maternal deaths occurred in multiparous women with previous cesarean section who had undergone lower segment cesarean section (LSCS) outside our institution and presented as primary PPH with acute hemorrhagic shock. Out of these, one case presented as atonic PPH while other three had traumatic PPH with hemoperitoneum in which one case was of rectus sheath hematoma and two were of broad ligament hematoma. Direct causes of death were hemorrhagic shock, septicemia and DIC. Two maternal deaths occurred within 24 hours of admission due to acute hemorrhagic shock followed by respiratory failure while two patients survived more than 24 hours of admission but died in the next 24 hours on postoperative day 2 due to septic shock and DIC followed by respiratory failure (Table 6).

**Table 6:** Maternal mortality-associated factors EOH: Emergency obstetric hysterectomy; PPH: Postpartum hemorrhage; DIC: Disseminated intravascular coagulation

Parameters	No. of Maternal Death (N=4)
Indication of EOH	
Atonic PPH	1
Traumatic PPH with hemoperitoneum	3
Cause of death	
Hemorrhagic shock	2
Septicemia	1
Hemorrhagic shock with DIC	1
Admission to death duration	
Within 24 hours	2
Within 48 hours	2

## Discussion

There is considerable variability in the incidence of emergency obstetrics hysterectomy in different countries and even among institutions. The overall incidence at our institution in the study of a two-year period was found to be 0.5941 per 100 deliveries. This was similar to Zhang et al. (0.63 per 100 deliveries) while higher than some of the Indian studies like Chawla et al. and Praneshwari et al. (0.08% and 0.0779%, respectively) [7-9]. Previous studies have indicated that women who have undergone a previous CS are at higher risk of EOH than women who have had only vaginal deliveries [7-9]. Likewise, we also found this to be the case for women admitted to our hospital, where the incidence of hysterectomy for previous CS was higher than for women with vaginal delivery (0.5553 per 100 deliveries and 0.03 per 100 deliveries, respectively). The reason for this can be an increase in the number of CSs performed, leading to an increase in abnormal placentation, placenta previa, and uterine scarring.

Most cases are of multiparous women of 25-35 years, which was similar to Praneshwari et al. and Sarah Kazi study [9,10]. Mode of delivery in our study was seen to be vaginal delivery (6.5%), cesarean (71.7%) and exploratory laparotomy (21.8%), and the results are consistent with Zhang et al. [7].

One of the primary benefits of EOH is its ability to rapidly control uncontrolled bleeding, which can occur due to complications like placenta accreta, uterine rupture, or severe postpartum haemorrhage. By removing the uterus, the source of bleeding is eliminated, preventing the loss of large volumes of blood and stabilizing the patient's condition. In the current scenario, EOH incidence due to uterine atony is declining from 42% to 29% due to the use of uterotonic and hemostatic agents, surgical techniques like internal iliac artery ligation while the incidence due to abnormal placentation is increasing from 25% to 41% [11,12]. Also, in multiparous women, prolonged labour and late referral are responsible for the high proportion of cases of uterine rupture [13]. The study in our institution showed that the majority 43.5% cases belonged to the morbidly adhered placenta, followed by 30.5% of rupture uterus and 26% of intractable PPH, which corresponds to Praneshwari et al., Pal et al., Mbakwa et al. and Forna et al. [9,14-16]. However, in Chawla et al., Sarah Kazi, and Bhattacharyya et al. [8,10,17], uterine atony was the major indication.

In performing emergency obstetric hysterectomy, subtotal hysterectomy is often done (60.9%) as compared to total hysterectomy (39.1%) because of the time factor as the quicker the operation is completed the better the outcome in a moribund patient. Similar results were seen in Shirodker et al. with 62% subtotal hysterectomies [18].

EOH can be a critical procedure in situations where the mother's reproductive health is compromised or when future pregnancies pose a significant risk to her health and well-being. However, it's important to acknowledge that emergency obstetric hysterectomy is a major surgical procedure and is not without risks. The procedure itself carries risks of intraoperative and postoperative complications, and the removal of the uterus has long-term implications for the woman's fertility and emotional well-being. Whenever possible, efforts should be made to explore alternative interventions and ensure the procedure is performed in a well-equipped facility with skilled healthcare professionals. In our study, the most frequent preoperative complication seen in EOH was severe anaemia found in 63% of cases. Common surgical complications include injury to the urinary tract (bladder injury seen in four cases and one case having ureteric injury in association with bladder injury) and other nearby structures, which may be avoided by better surgical training or multi‑specialist approach involving urological or general surgeons. Almost all patients needed ventilator support leading to maternal morbidity. Postoperatively, acute hemorrhagic shock was seen in more than half of the patients, followed by DIC, septicemia, wound infection, acute renal failure, hepatic encephalopathy, and psychosis. Similar complications were reported by Zhang et al., Sarah Kazi and Mbakwa et al. [7, 10, 15].

Maternal mortality is 8.7% in our study which shows that EOH plays a crucial role in reducing maternal morbidity and mortality by providing a timely and effective solution, and results were comparable with Praneshwari et al. reporting no mortality and Mbakwa et al. with 4% mortality [8, 10]. However, there are also some studies showing higher mortality rates like Sarah Kazi and Chawla et al. reporting 19% and 17.9% mortality rates, respectively [8,10]. Due to the lesser number of maternal and perinatal deaths, we were unable to draw valid conclusions on mortality-associated factors. Also as all patients who died were delivered outside the institution, less data is available to analyze the predisposing factors leading to mortality. However, in our study, the association of maternal death is mainly due to PPH (atonic or traumatic) and acute hemorrhagic shock with death within 24-48 hours of admission.

To the best of our knowledge, this is the preliminary study in our institute that compares and brings out data on the incidence, indications and outcomes of EOH. However, the limitation of this study was its retrospective design and data collected from a single institution. Also, we have not shown other primary measures which have reduced the necessity of EOH. Furthermore, most women were grossly anaemic on arrival and had no prior antenatal records altering analysis of EOH complications.

## Conclusions

EOH is an unwanted but necessary emergency in obstetrics. Still, it is a lifesaving procedure in many catastrophic conditions of obstetrics, although it curtails the future childbearing capacity of females. Most of the complications are attributed to its indications and underlying disease rather than surgical procedure. Previous cesarean sections and associated conditions are the most common indication for EOH. Extensive training in this rare skill can save the lives of mothers, so this should be a part of postgraduate training.
